# Sepsis Related Mortality Associated with an Inflammatory Burst in Patients Admitting to the Department of Internal Medicine with Apparently Normal C-Reactive Protein Concentration

**DOI:** 10.3390/jcm11113151

**Published:** 2022-06-01

**Authors:** Ronnie Meilik, Hadas Ben-Assayag, Ahuva Meilik, Shlomo Berliner, David Zeltser, Itzhak Shapira, Ori Rogowski, Ilana Goldiner, Shani Shenhar-Tsarfaty, Asaf Wasserman

**Affiliations:** 1 Department of Internal Medicine “C”, “D”, & “E”, Tel Aviv Medical Center, Sackler Faculty of Medicine, Tel Aviv University, Tel Aviv 64239, Israel; ronniemeilik@gmail.com (R.M.); hadasba@tlvmc.gov.il (H.B.-A.); berliners@tlvmc.gov.il (S.B.); dzeltser@tlvmc.gov.il (D.Z.); shapira@tlvmc.gov.il (I.S.); orir@tlvmc.gov.il (O.R.); asafw@tlvmc.gov.il (A.W.); 2 Clinical Performances Research and Operational Unit, Tel Aviv Medical Center, Sackler Faculty of Medicine, Tel Aviv University, Tel Aviv 64239, Israel; ahuvawm@tlvmc.gov.il; 3 Laboratory Medicine, Tel Aviv Medical Center, Sackler Faculty of Medicine, Tel Aviv University, Tel Aviv 64239, Israel; ilanag@tlvmc.gov.il

**Keywords:** C-reactive protein, inflammation, mortality causes

## Abstract

Background: Patients who are admitted to the Department of Internal Medicine with apparently normal C-reactive protein (CRP) concentration impose a special challenge due the assumption that they might not harbor a severe and potentially lethal medical condition. Methods: A retrospective cohort of all patients who were admitted to the Department of Internal Medicine with a CRP concentration of ≤31.9 mg/L and had a second CRP test obtained within the next 24 h. Seven day mortality data were analyzed. Results: Overall, 3504 patients were analyzed with a mean first and second CRP of 8.8 (8.5) and 14.6 (21.6) mg/L, respectively. The seven day mortality increased from 1.8% in the first quartile of the first CRP to 7.5% in the fourth quartile of the first CRP (*p* < 0.0001) and from 0.6% in the first quartile of the second CRP to 9.5% in the fourth quartile of the second CRP test (*p* < 0.0001), suggesting a clear relation between the admission CRP and in hospital seven day mortality. Conclusions: An association exists between the quartiles of CRP and 7-day mortality as well as sepsis related cause of death. Furthermore, the CRP values 24 h after hospital admission improved the discrimination.

## 1. Introduction

The admission of patients to the Department of Internal Medicine with apparently normal C-reactive protein (CRP) concentration is a clinical challenge due to the possibility that clinicians might assume that these patients do not harbor a significant inflammatory response. However, the inflammatory response could burst-in later. “Inflammatory burst” is the rapid release of pro-inflammatory cytokines/mediators. Previous studies have shown that any inflammatory process could put the patient at great risk due to tissue damage or necrosis mechanisms [1,2]. Therefore, repeated measures of inflammatory biomarkers are more informative than looking at a single snapshot. We have recently shown that patients who are admitted with very low CRP concentrations do not necessarily present a benign course of their disease [3]. In addition, we could show that a follow-up CRP test could add significant prognostic information to the medical team [4,5,6,7,8]. In fact, a second CRP test could single out those individuals who are at an increased risk of death during hospitalization [9].

We conducted a retrospective study in a cohort of patients who were admitted to the Departments of Internal Medicine with apparently normal C-reactive protein concentration, in whom, a short-term follow-up CRP test was performed. The specific aim of this study was to determine the relation of 7-day mortality to the CRP values in the first 24 h after admission. This information is relevant for the usefulness of doing a follow-up CRP in individuals in whom the treating physician might have an impression of a non-alarming medical condition.

## 2. Patients, Controls and Methods

### 2.1. The Patients

We used the MDClone system to retrieve information regarding the patients who were admitted to one of our nine Departments of Internal Medicine at the Tel-Aviv Sourasky Medical Center, a tertiary 1050-bed university affiliated medical center serving a population of about 500,000 residents of the city of Tel-Aviv, Israel. Included were patients who presented with a CRP concentration of ≤31.9 mg/L and had a second CRP test obtained within 24 h thereafter. Since no postmortem sections were performed in those individuals who did not survive the first week of hospitalization, the medical records of those patients were manually reviewed on an individual basis in order to determine the presumed cause of death in an as accurate a way as possible.

### 2.2. The Method to Determine the CRP Cutoff

The method of determining the cutoff CRP concentration was based on data that were available in the Tel-Aviv Medical Center Inflammation Survey (TAMCIS) as previously described [10]. In brief, a CRP concentration of 31.9 mg/L was actually the upper limit of a mean CRP + three S.D. obtained from 17,214 apparently healthy individuals who participated in our health-screening program [11,12,13,14]. Therefore, only hospitalized patients with the first CRP measured to be lower or equal to 31.9 mg/L were presently included.

### 2.3. The MDClone System

Data were retrieved using MDClone (mdclone.com), a query tool that provides the comprehensive patient-level data of wide-ranging variables in a defined period around an index event. Data were collected for all patients over 18 years old hospitalized between June 2007 and September 2020.

### 2.4. Laboratory Methods

Wide-range CRP (wrCRP) was measured by ADVIA 2400 Siemens Healthcare Diagnostics Inc., Tarrytown, NY 10591-5097 USA using a Latex enhanced immunoturbidimetric method [15].

### 2.5. Review of Death Causes

Cause of death was determined by reviewing individual record files. After a patient’s death, the treating medical team had thoroughly recorded their diagnosis by relying on different findings and the patient’s clinical picture during hospitalization. Sepsis, in particularly, was determined as the cause of death when the patients presented with multiorgan failure including shock and cause of death in a picture implying sepsis.

### 2.6. Statistical Methods

Categorical variables were reported as numbers and percentages. Continuous variables were evaluated for normal distribution and reported as the mean and standard deviation (SD) or as the median and interquartile range (IQR). Subgroup analysis of the first and second CRP levels was conducted using the Mann–Whitney test. The chi squared test or Fishers’ exact test were used to compare the categorical variables among patients who survived the first 7 days of admission and those who did not. The receiver operating characteristic (ROC) curve analysis was used to evaluate the serial CRP measurements as the predictor of 7 day mortality. A time-dependent COX regression was used to evaluate the association between each of the CRP measurements and in-hospital mortality. Age, sex, and either the first or second CRP measurements were included in the analysis. A two-tailed *p*-value < 0.05 was considered as statistically significant. IBM SPSS (IBM Corp. Released 2013. IBM SPSS Statistics for Windows, Version 25.0. Armonk, NY: IBM Corp.) was used for all statistical analyses.

### 2.7. Ethics Committee Approval

The Tel-Aviv Sourasky Medical Center Institutional Review Board (0491-17-TLV) approved the study.

## 3. Results

Overall, 3504 inpatients met the inclusion criteria. The mean age was 64.3 (18.5) years. The mean first and second measurements of CRP were 8.8 (8.5) and 14.6 (21.6) mg/L, respectively. The characteristics of the patients are described in Table 1.

CRP is known to be affected by a various factors, therefore, subgroup analysis of the first and second CRP levels was conducted (Table 2). Female gender, diabetes mellitus, hypertension, and ischemic heart disease were associated with higher levels of both the first and second CRP measurements in our cohort.

In Table 3, we present the results of the first CRP divided into quartiles. It can be seen that the seven day mortality rates increased from 1.7% in the first CRP quartile to 7.8% in the fourth one (chi-square statistics was 37.6, *p* < 0.0001).

In the same table, we show the percentage of mortality in the different quartiles of the second CRP test that was taken within 24 h from admission. Again, the seven day mortality rates increased according to the CRP increment being 0.5% in the first quartile as opposed to 9.5% in the fourth one (chi-square statistics was 85.0, *p* < 0.0001).

Therefore, while the death percentage was 4.6 times higher in the fourth as opposed to the first quartile of the first CRP test, this difference was 19 times higher in the fourth as opposed to the first quartile of the follow-up CRP test (second CRP measurement, right side of Table 1).

The lowest mortality rate was found in patients with the second measurement of CRP in the first quartile. These patients who arrived with a CRP below 31.9 mg/L and whose CRP concentrations remained minimal on the second day had a 3.4 times less mortality rate. Patients in the first CRP quartile of the first measurement presented a 0.5% mortality rate. Distinctly different, patients in the first CRP quartile of the first measurement demonstrated a 1.7% mortality percentage.

The negative predictive value of patients admitting to the Department of Internal Medicine with the first CRP <31.9 and second measurement of CRP in the lowest quartile (CRP <2 mg/L) was 0.0046.

The area under the ROC curve (AUC) when using the first CRP measurement as the predictor of 7 day mortality was 0.639 (0.599–0.680) *p* < 0.001. This AUC increased to 0.731 (0.696–0.766) *p* < 0.001 when using the second measurement of CRP.

**Figure 1:**
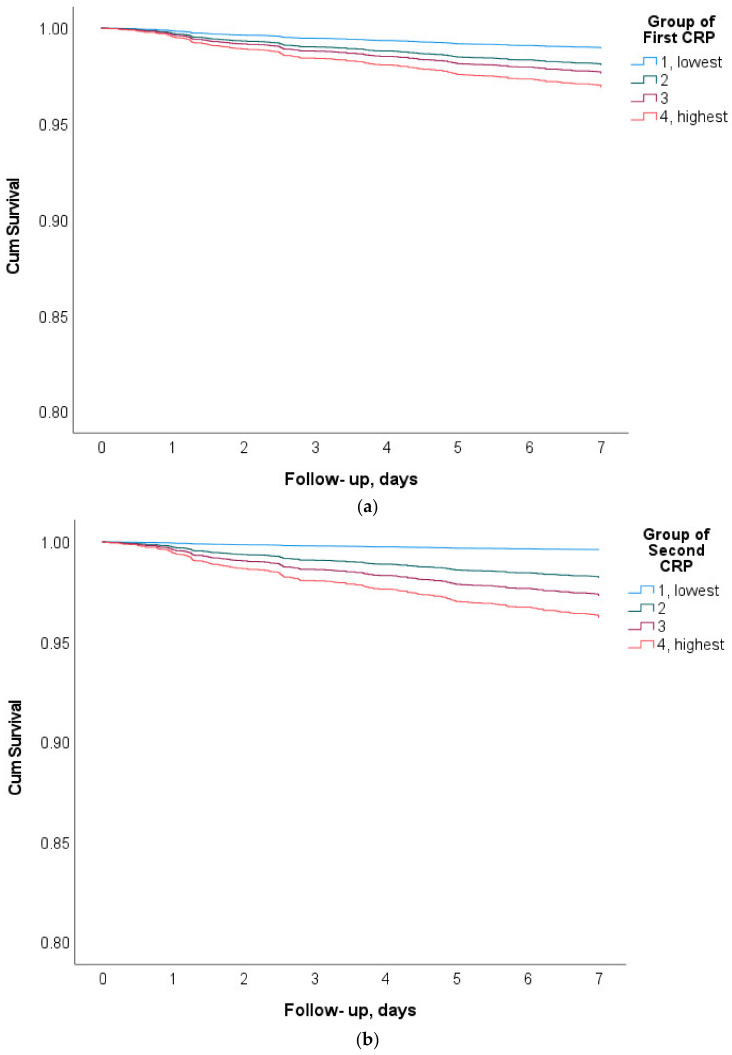
The survival plots for the first (**a**) and second (**b**) CRP quartiles (one is the lowest quartile, four is the highest). Log-rank mantel-Cox) < 0.001 for both.

Furthermore, the Cox regression using age, gender, and the first and second CRP quartiles together confirmed that the age and quartile of the second CRP measurements had a significant effect on mortality (hazard ratios (exp(b) being 1.08 for age, and 5.8, 10.0, and 14.1 for quartiles 2, 3, and 4, respectively, of the follow-up CRP test (*p* ≤ 0.001 for all))) (Figure 1).

Of special interest is our finding that the sepsis cause of deaths increased in a dose dependent manner with the quartiles of the first and second CRP. Patients with an extreme low level of CRP (first quartile) not only had aa better survival rate, but also had a lower risk of mortality from sepsis compared to patients at the highest quartile of either the first or second measurement of CRP (6.7% and 0% of mortality from sepsis for patients at the first quartile compared to 54.4% and 47.0% at the fourth quartile of the first and second CRP measurements, Table 3).

## 4. Discussion

To the best of our knowledge, this is the first study to explore the causes of death in patients who were admitted to the Department of Internal Medicine with apparently normal CRP concentration and in whom a follow-up CRP test was obtained within 24 h from admission. It was found, indeed, that despite presenting with a relatively low-grade inflammatory response that could potentially be observed in an apparently healthy population, these individuals might harbor severe and potentially lethal medical conditions. This concept was recently described by our group in patients who were admitted to the hospital with CRP concentrations that were below the detection level of the wr-CRP test [3].

We focused on the reasons of death in patients who were admitted to the Department of Internal Medicine with apparently normal CRP concentration and presented various degrees of inflammatory bursts by performing a follow-up CRP test within the first 24 h of their hospitalization. This study is especially relevant since it is known that the inflammatory response is not only a marker for the severity of the disease, but is involved in pathophysiological changes that could have deleterious effects, especially if exaggerated. This has clearly been shown in several clinical models such as in patients with ST-elevation myocardial infarction (STEMI) [16,17,18] as well as in the recent COVID-19 pandemic [19,20,21]. What we found is a gradual correlation between the intensity of the inflammatory response and the probability of death from different medical conditions within a relatively short period. These findings highly suggest not relying on a single apparently normal C-reactive protein CRP concentration upon admission to a medical facility, but insisting on at least one, if not more than one, additional test to follow.

The notion that the inflammatory response is a dynamic one and that the acute phase response has a well-established course is well-known [22]. However, due to economical availability as well as organizational difficulties, clinicians often do not perform repeated tests, and this is especially true in conditions where the results of the CRP test are not high. In fact, facing an individual with high CRP concentrations presents no special dilemmas to the clinician with regard to the question oof whether the patient has a significant inflammatory response or not. The main problem concentrates around those who seek medical care and do not present a heightened inflammatory response.

Although this article focused on CRP, it is important to mention that other markers could be used by clinicians to evaluate an inflammatory process such as hemoglobin, white blood cell count, fibrinogen, cytokines, chemokines, complement factors, adhesion molecules, and the blood sedimentation rate [23,24,25,26,27,28].With the advanced data-driven machine learning methods, we assumed that in the near future, it would be possible to handle multiple biomarkers simultaneously to gain a much more accurate mortality prediction [29,30,31].

There is no agreement in the medical literature or between researchers of what an apparently normal CRP concentration means. In an apparently healthy population, low risk for cardiovascular disease is defined as CRP <1 mg/L and high risk as >3 mg/L [32]. In order to cover the vast majority of apparently healthy individuals, we chose the cutoff of mean CRP plus three standard deviations, which was equal to 31.9 mg/L in our cohort of apparently healthy individuals who attended a routine annual health-screening program and had no signs or symptoms of an active inflammatory disease including specific questions that were asked regarding such an eventual active inflammatory disease/disorder. The details of this cohort have been extensively described in the past [11,12,13,14]. We then decided arbitrarily to define the apparently normal C-reactive protein CRP concentration as values that were below this upper + three standard deviations, although one could argue that this is too a high level. Nevertheless, we made this decision to cover almost all CRP concentrations that can be detected in a population that does not seek medical assistance for an acute illness.

Of special interest was the finding that the correlation between the intensity of the inflammatory burst and the seven day mortality was not limited to infectious conditions but included different acute medical conditions including stroke, respiratory failure, sudden cardiac arrest, and others (see Supplementary Table S1). In fact, inflammation-related clinical deterioration has been described in diseases and disorders that are not caused by infective organisms [33,34,35,36]. In addition, we showed that the lack of a follow-up inflammatory burst is associated with significantly less mortality. Although one may assume that the lack or minimal inflammatory burst is only a reflection of a less severe disease/disorder, looking at the evolution of the anti-inflammatory treatments given in the COVID-19 pandemic, we could also raise the possibility that the limited inflammatory burst contributed to a better prognosis. In fact, the inflammatory burst is not necessarily an innocent bystander, and early anti-inflammatory interventions could have a beneficial role in the evolution of the disease.

Finally, we draw attention to the fact that sepsis was the leading reason for seven day mortality in this cohort. This is especially relevant for daily practice, since we included a cohort of all comers and not necessarily those with infectious diseases. Although this observation should be further investigated, the appearance of a significant inflammatory burst in the Department of Internal Medicine should raise the possibility of an ongoing acute infection.

Some limitations of this study should be taken into consideration. The main limitation was that this was a retrospective observational study. In addition, sorting out a certain group of patients that have doubtful criteria for sepsis diagnosis and CRP below a certain threshold might impose a selection bias. However, all patients who met the inclusion criteria were admitted to the hospital in the context of suspicious clinical images, regardless of their CRP values, and all CRP measurements were conducted in a single lab using the same laboratory method. In addition, some possible confounders of CRP levels were not recorded in our database such as race. In our subsequent studies, we wish to evaluate these prospectively. Another limitation was the possibility of losing the significance of the association between the CRP values and the mortality rates due to other confounders in the regression model.

We can conclude that the appearance of a significant inflammatory burst in patients who are admitted to the Department of Internal Medicine is associated with a worse prognosis, and the possible existence of an ongoing acute infection should be taken into consideration. A first apparently normal CRP concentration should be followed by additional tests to exclude serious medical conditions with poor prognosis. Clinicians should not make any firm prognostic conclusions before additional tests are performed in these special populations.

## Figures and Tables

**Table 1:** The characteristics of the patients.

Total Population	N = 3504
Gender (% of males)	1.7
Age (Years: mean ± SD)	64.3 ± 18.5
Hypertension, %	26.7
Diabetes, %	14.4
Ischemic heart disease, %	13.1
Dyslipidemia, %	11.3
CVA %	2.9

**Table 2:** The subgroup analysis of the first and second CRP measurements.

Factor	First CRP (Median, IQR)			Second CRP (Median, IQR)		
	With	Without	*p* Value	With	Without	*p* Value
Gender, (Female)	6.24 (1.6–14.0)	5.37 (1.4–13.8)	<0.001	8.45 (2.3–19.5)	7.74 (2.0–19.3)	<0.001
Diabetes Mellitus	7.54 (2.4–15.7)	5.50 (1.3–13.8)	<0.001	9.48 (3.2–20.2)	7.8 (2.0–19.3)	<0.001
Hypertension	6.91 (2.-15.0)	5.38 (1.3–13.8)	<0.001	9.09 (2.9–19.9)	7.7 (1.9–7.7)	<0.001
Dyslipidemia	5.57 (1.7–13.2)	5.81 (1.4–14.3)	0.684	7.47 (2.3–17.6)	8.15 (2.1–19.7)	<0.001
Ischemic heart disease	6.65 (1.9–14.9)	5.66 (1.4–14.0)	<0.001	8.42 (2.4–19.3)	8.02 (2.1–19.4)	0.008

**Table 3:** The number of patients who died within 7 days according to the quartiles of the first and second CRP measurements.

First CRP Measurement					Second CRP Measurement				
Quartile	n	CRP, mg/L	Deaths within 7 Days n, (%)	Sepsis Related Deaths, n, (%)	Quartile	n	CRP, mg/L	Deaths within 7 Days n, (%)	Sepsis Related Deaths, n, (%)
1	878	0.6 (0.5)	15, (1.7%)	1, (6.7%)	1	879	0.85 (0.6)	4, (0.5%)	0, (0%)
2	874	3.4 (1.2)	33, (3.8%)	6, (18.2%)	2	873	4.4 (1.6)	27, (3.1%)	6, (22.2%)
3	876	9.5 (2.4)	47, (5.4%)	19, (40.4%)	3	876	12.2 (3.1)	49, (5.6%)	18, (36.7%)
4	876	21.5 (5.1)	68, (7.8%)	37, (54.4%)	4	876	40.9 (29.3)	83, (9.5%)	39, (47.0%)
Sum	3504	8.8 (8.5)	163, (4.65%)	63, (38.7%)	Sum	3504	14.6 (21.6)	163, (4.65%)	63, (38.7%)

## Data Availability

Data will be made available upon reasonable request.

## Supplementary Materials

The following supporting information can be downloaded at: https://www.mdpi.com/article/10.3390/jcm11113151/s1, Table S1: Cause of death by quartiles of 1st and 2nd CRP measurements.
