# Supplementary material for: Sepsis Related Mortality Associated with an Inflammatory Burst in Patients Admitting to the Department of Internal Medicine with Apparently Normal C-Reactive Protein Concentration
Source: J Clin Med. 2022 Jun 1;11(11):3151. doi: 10.3390/jcm11113151 (PMC9181046; doi:10.3390/jcm11113151)
Supplement: Supplementary file 1 [file jcm-11-03151-s001.zip › jcm-1604400-supplementary.pdf]

Supplementary Table S1. Cause of death by quartiles of 1st and 2nd CRP measurements.

**Quartile of 1st CRP test**	**Cause of death**	**n**
1	cardiogenic shock	1
	Intracranial hemorrhage	8
	Ischemic stroke	2
	sepsis	1
	sudden cardiac arrest	2
	unknown	1
2	advanced metastatic disease	1
	anoxic brain damage	1
	cardiogenic shock	2
	Intracranial hemorrhage	10
	multiple organ failure	3
	Respiratory failure	5
	sepsis	6
	status epilepticus	1
	sudden cardiac arrest	3
	unknown	1
3	cardiogenic shock	1
	CHF exacerbation	2
	drowning	1
	hemodynamic failure	1
	hemorrhagic shock	1
	Hepatic encephalopathy	1
	Intracranial hemorrhage	1
	Ischemic stroke	3
	multiple organ failure	3
	pulmonary embolism	1
	Renal failure	1
	Respiratory failure	2
	sepsis	19
	sudden cardiac arrest	5
	unknown	5
4	cardiogenic shock	2
	CHF exacerbation	2
	Intracranial hemorrhage	1
	Ischemic stroke	1
	multiple organ failure	5
	Respiratory failure	4
	rhabdomyolysis	1
	sepsis	37
	sudden cardiac arrest	10
	unknown	5
	Grand Total	163

Quartile of 2nd CRP test	Cause of death	n
1	cardiogenic shock	1
	Intracranial hemorrhage	1

	sudden cardiac arrest	1
	unknown	1
2	advanced metastatic disease	1
	anoxic brain damage	1
	cardiogenic shock	1
	Intracranial hemorrhage	1
	Ischemic stroke	1
	multiple organ failure	4
	Respiratory failure	5
	sepsis	6
	sudden cardiac arrest	5
	unknown	2
3	cardiogenic shock	2
	Congestive Heart Failure exacerbation	2
	hemodynamic failure	1
	hemorrhagic shock	1
	Hepatic encephalopathy	1
	Intracranial hemorrhage	8
	Ischemic stroke	2
	multiple organ failure	3
	pulmonary embolism	1
	Renal failure	1
	Respiratory failure	4
	sepsis	18
	status epilepticus	1
	sudden cardiac arrest	2
	unknown	2
4	cardiogenic shock	2
	Congestive Heart Failure exacerbation	2
	drowning	1
	Intracranial hemorrhage	10
	Ischemic stroke	3
	multiple organ failure	4
	Respiratory failure	2
	rhabdomyolysis	1
	sepsis	39
	sudden cardiac arrest	12
	unknown	7
	Grand Total	163
